# Does Impaired Glymphatic Drainage Cause Glymphedema? A Review Tailored to Neurocritical Care and Neurosurgery

**DOI:** 10.1007/s12028-021-01224-1

**Published:** 2021-06-10

**Authors:** Paul T. Akins, Kern H. Guppy

**Affiliations:** Department of Neurosurgery, Kaiser Permanente, Sacramento, CA USA

**Keywords:** Glymphatic system, Hydrocephalus, Subarachnoid hemorrhage, Hydrocephalus (normal pressure), Brain injuries (traumatic), Ischemic stroke, Meninges, Aquaporins, Decompressive craniectomy, Lymphatic system, Astrocytes, Subdural effusion, Neurovascular coupling

## Abstract

Research into the glymphatic system reached an inflection point with steep trajectory in 2012 when it was formally recognized and named, but the historical roots for it are solid and deep, dating back to pioneers such as Cushing, Weed, and Dandy. We provide an overview of key discoveries of the glymphatic system, which promotes bulk flow of fluid and solutes throughout the brain parenchyma. We also discuss the lymphatic drainage of the central nervous system. Evidence is building that failure of the glymphatic system causes glymphedema in patients commonly managed by neurocritical care and neurosurgery specialists. We review research supporting this for decompressive craniectomy, subarachnoid hemorrhage, and normal-pressure hydrocephalus. We argue that it is time for a paradigm shift from the traditional model of cerebrospinal fluid circulation to a revised model that incorporates the glymphatic pathway and lymphatic clearance. These recent breakthroughs will inspire new therapeutic approaches to recognize, reverse, and restore glymphatic dysfunction and to leverage this pathway to deliver brain-wide therapeutics.

## Background

Basic science and clinical studies are rapidly expanding our knowledge of the glymphatic system. For example, a PubMed search of “glymphatic system” yielded five citations in 2013, 28 in 2016, and 149 in 2019. The goal of this article is to raise awareness and stimulate interest among colleagues, as evidence is mounting that there is a dynamic interplay between neurologic and neurosurgical diseases and the glymphatic system. We will argue that traditional models for hydrocephalus and cerebral edema warrant revision to recognize the central role that the glymphatic system plays in both the circulation of cerebrospinal fluid (CSF) and the clearance of interstitial fluid (ISF) and macromolecules as well. We will review basic and clinical research that shows how both diseases and treatments may cause glymphedema, which we define as abnormal craniospinal fluid collections due to impaired glymphatic drainage.

In *The Practice of Emergency and Critical Care Neurology*, published in 2010 [1], Dr. Wijdicks concisely presents the classic teaching of CSF circulation that most readers (authors included) learned early in their training:

Cerebrospinal fluid is produced in the choroid plexus of the lateral ventricle; circulated throughout a system with critical passages at the foramen of Monro, third ventricle, and aqueduct of Sylvius; and absorbed through arachnoid villi. Any obstruction of flow at these sites causes increased hydrostatic pressure in a matter of hours.

The classic model provides a practical framework toward diagnosis and treatment of patients with hydrocephalus; however, there is no mention of the glymphatic system. The reason for this is simple, as the term was recently introduced in 2012 [2].

What is the glymphatic system? Iliff et al. [2] gave this name to the “brain-wide pathway for fluid transport, which includes para-arterial influx of subarachnoid CSF into the brain interstitium, followed by the clearance of interstitial fluid along large-caliber draining veins.” The classic model does not incorporate other sources of CSF production beyond the choroid plexus. This traditional model also overlooks the craniospinal lymphatic system and perineural pathways for CSF drainage and their connection with the glymphatic pathway.

We begin with a review of key historical work and recent basic research then conclude with a discussion of emerging clinical implications for neurocritical care and neurosurgery specialists. Those already familiar with the glymphatic system may wish to skip ahead to the clinical section.

## Sleeping Giants: Early Research Identified Key Elements of the Glymphatic System

Many readers are familiar with both the pioneering work and academic rivalries [3] of Cushing, Weed, and Dandy regarding CSF pathways and hydrocephalus. Dr. Lewis Weed published research conducted in the laboratory of Dr. Harvey Cushing in 1914. He placed strong emphasis on the arachnoid villi as the primary site for CSF resorption [4]. In his posthumous 1946 paper on external hydrocephalus [5], Dr. Walter Dandy acknowledged the view of Weed that cerebrospinal “fluid passes into the longitudinal sinus by way of the Pacchionian granulations or other preformed stomas in the walls of the longitudinal sinus (this is the conception of Weed).”

Yet the writings of Dandy make it clear that he strongly disagreed that the arachnoid villi were the main site for CSF resorption: “Cerebrospinal fluid absorbs in the subarachnoid space (directly into the capillaries in every part of the subarachnoid space), and not into the dural sinuses or through special structures such as the Pacchionian granulations” [6]. This observation is a key step toward our current understanding of the glymphatic system.

**Fig. 1:**
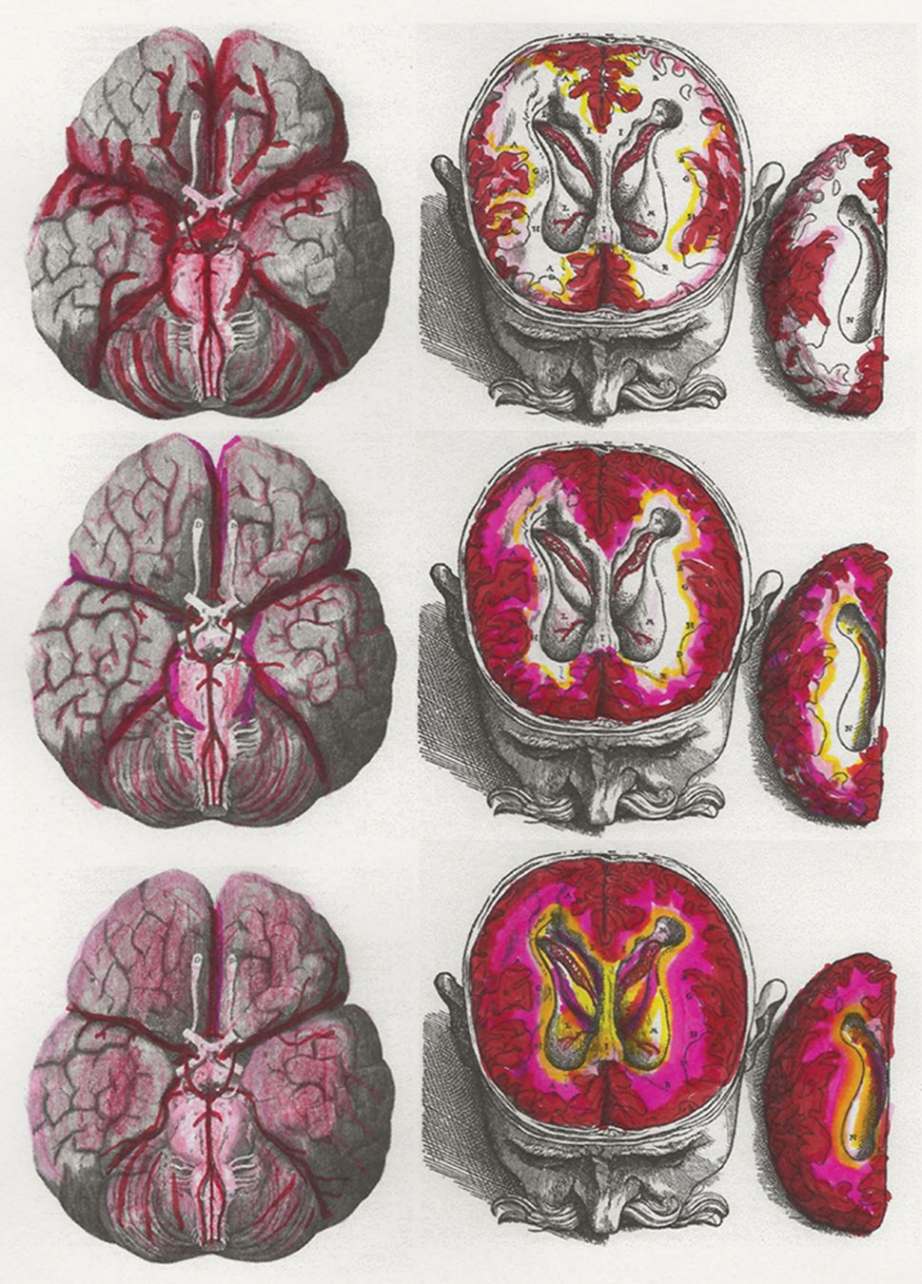
Cerebrospinal fluid (CSF) tracers have a brain-wide distribution along the subarachnoid space and perivascular spaces (Virchow–Robin spaces) through the glymphatic pathway [2]. Dandy used India ink as a CSF tracer to show this rapid redistribution from the basal cisterns into the subarachnoid space adjacent to the circle of Willis and along its major arterial branches (top row). The glymphatic pathway was recently confirmed in humans by observing the distribution of a gadolinium CSF tracer (gadobutrol) from the subarachnoid space deep into the brain parenchyma using serial magnetic resonance imaging (23). The gadobutrol CSF tracer enters the cortical mantle by 6–12 h (middle row). By 24–48 h, it is found deep within brain tissue (bottom row). The intrathecal gadobutrol is completely cleared at 4 weeks. The red, pink, yellow, and white color scale qualitatively indicates tracer enrichment. Adapted from Ringstad et al. [23] by using the 1664 engravings in *Cerebri Anatome* by Sir Thomas Willis (left column) and the 1555 engravings in *De Humani Corporis Fabrica* by Andreas Vesalius (middle and right columns). We encourage readers to visit the link to the supplemental video kindly provided by Ringstad and colleagues (https://www.ncbi.nlm.nih.gov/pmc/articles/PMC6124518/bin/jciinsight-3-121537-s253.mp4)

Dandy grounded his opinion on experiments conducted in dogs [7]:

The circulation of cerebrospinal fluid can be strikingly demonstrated by substituting India-ink for cerebrospinal fluid in an anaesthetized dog.… Almost immediately all the cisternae are filled with ink; the cerebellar subarachnoid space also rapidly fills, owing to its intimate relationship with the cisterna magna. Gradually filaments of ink fill the sulci over all surfaces of both cerebral hemispheres; the sulci radiate from the cisterna and appear to anastomose over the cerebral hemispheres (Fig. 1, top row).

From these experiments, he observed that “fluid is distributed along the major branches of the subarachnoid space which accompany the blood-vessels and traverse the sulci between the cerebral convolutions.” His observation regarding the close association of cerebral arteries and CSF circulation is a second key observation.

### Weed Describes “The Accessory or Lymphatic Pathway of (Cerebrospinal Fluid) Absorption” (1914)

Although the work of Weed to identify and promote the role of the arachnoid villi (also known as Pacchionian or arachnoid granulations) in CSF absorption is widely known, another early observation still lingers in obscurity. In addition to CSF absorption via arachnoid villi, he described “the accessory or lymphatic pathway of (CSF) absorption” [4]. Using a ferrocyanide suspension infused into the spinal subarachnoid space under pressure (50 mm Hg), followed by delayed examination of the head of the animal subject (dogs and cats) after arterial formalin infusion, blue cords caused by ferrocyanide staining were observed along (1) the carotid sheath, (2) the lymph nodes at the skull base and neck, (3) the perineurium of the cranial nerves, (4) the cribriform plates and nasal mucosa, (5) the ocular episcleral space, and (6) the perineurium along the anterior and posterior spinal nerve roots connecting to lymphatic channels. He concluded that “Our preparations strongly indicate an escape of the (cerebro-) fluid outward along the cranial nerves, especially in the perineural spaces. From these it becomes taken up by the lymph channels.”

These early publications by Dandy and Weed report bookend discoveries about the entry and exodus of CSF and CSF tracers via the glymphatic pathway.

### Cushing and Weed Identify a Dual Source of CSF

Dr. Harvey Cushing understood that CSF was a “circulatory medium” for the brain that served as a “third circulation” [8]. He posed key questions in 1914 that are now being answered a century later [9]: “Are there lymph channels in the brain, and if not, how does the central nervous system dispose of its products of tissue waste?” and “If there are cerebral lymphatics, do they discharge into the subarachnoid spaces…?” He cited Weed’s intrathecal ferrocyanide experiments (see above). Precipitated Prussian blue staining extended from the subarachnoid space, along cerebral sulci, around arteries and veins, and on down to the capillary level. Cushing reasoned that fluid in this perivascular space would flow out of the brain parenchyma toward the subarachnoid space and constitute a “dual source” of CSF under physiologic conditions, as supraphysiologic intrathecal pressures were necessary to backfill this system.

Weed was confident that this dual source of CSF served an important role [10]: “Nervous tissue lacks entirely the lymphatic system; it would appear that its place is taken by the perineuronal, pericapillary, and perivascular system with its contained fluid, and that this fluid is poured into the subarachnoid space, where it mixes with the fluid from the choroid plexuses.” He postulated that “[t]his whole accessory fluid system of the cerebro-spinal axis…undoubtedly possesses an active function in maintaining the metabolic exchange and elimination of the nerve cells.”

With that historic overview, we will now shift to recent research that confirms and builds on these pioneering discoveries.

### Light and Electron Microscopy Studies Confirm a Perivascular Circulation of CSF from the Subarachnoid Space Into Brain Parenchyma

**Fig. 2:**
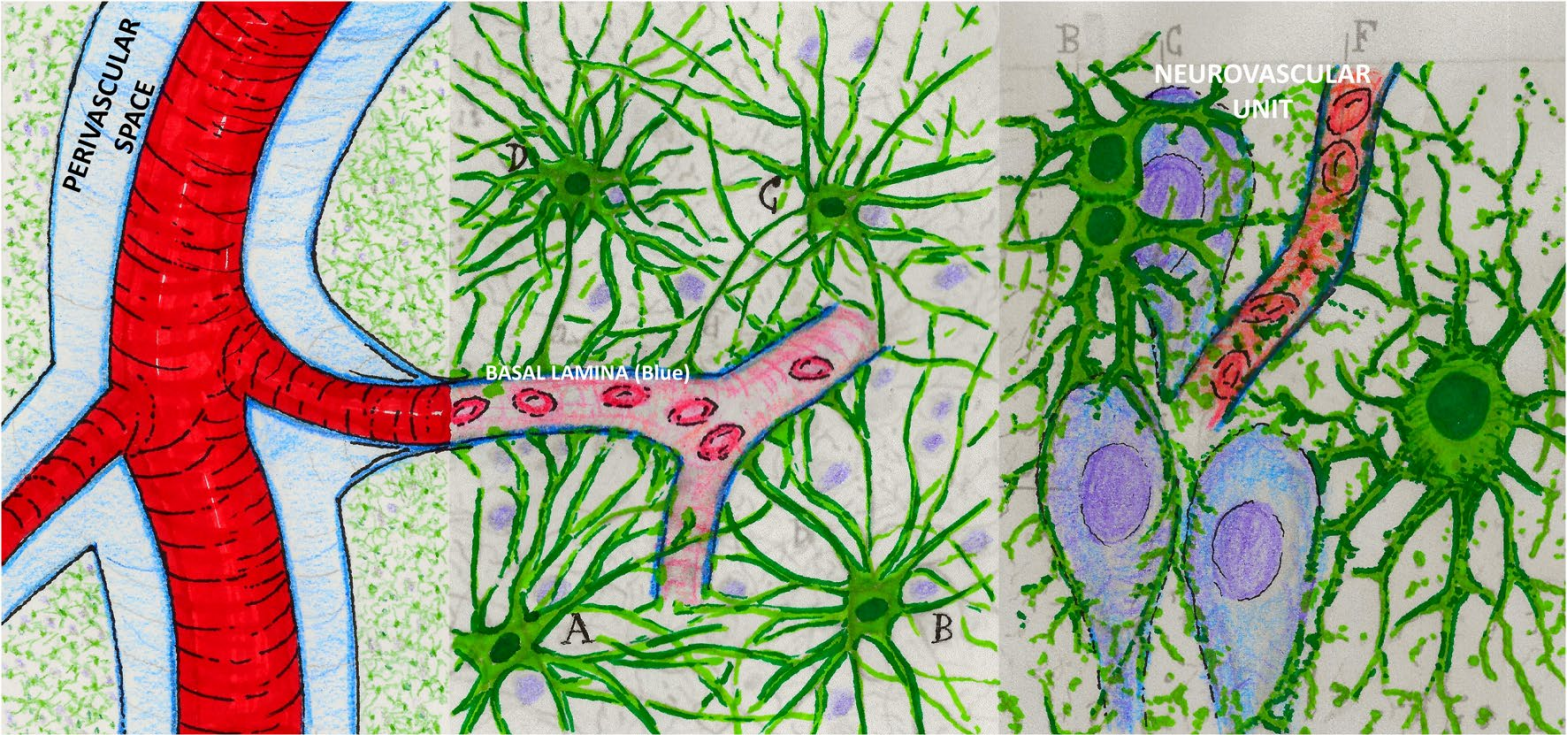
The perivascular space (light blue) diminishes in size as the vessels branch to smaller calibers and becomes continuous with the basal membrane (dark blue) that surrounds the capillaries (left panel). The basal membrane is a porous gel composed of type IV collagen, fibronectin, laminins, and proteoglycans. The outer layer of the perivascular space around blood vessels in the brain is lined with end-feet of astrocytes (A, B, C, D; green) and called the glial limitans perivascularis (middle panel). The neurovascular unit is composed of the closely associated neurons (purple), astrocytes (green), and blood vessels (red) (right panel). Interstitial fluid and solutes in brain tissue can enter the perivascular spaces by aquaporin 4 channels highly expressed in the end-feet and through the 20-nm intercellular clefts between end-feet. The middle and right panels are adapted from drawings by Santiago Ramón y Cajal (circa 1906, the year he received the Nobel Prize in medicine). He exaggerated the size of the astrocytes to draw attention to their importance

These initial discoveries lay largely dormant until the 1970’s. Hakim et al. [11–13] described patients with normal-pressure hydrocephalus (NPH) and proposed that the brain could be modeled as “an open cell sponge made of viscoelastic material.” Researchers confirmed that the brain had prominent extracellular spaces that accommodated considerable movement of large molecules. The extracellular space was modeled as “a highly hydrated gel consisting of a matrix of acid mucopolysaccharides and proteoglycans, and an aqueous phase similar in composition to cerebrospinal fluid” [14]. This biophysical model resonated well with Hakim’s insights and our current understanding of the continuity of perivascular spaces with the basal membrane of the cerebral microvasculature (Fig. 2).

Cerebrospinal fluid tracer experiments were repeated by using various large-molecule agents, such as Blue Dextran 2000, horseradish peroxidase (43 kDa), and yellow Microphil, across seven mammalian species, including nonhuman and human primates [14–16]. The distribution of these tracers was more rapid than predicted by simple diffusion and indicated bulk flow along the perivascular spaces. These perivascular spaces had been observed in the 1850’s and termed “Virchow–Robin” spaces [17, 18]. Light and electron microscopy confirmed distribution of CSF tracers down to the basal laminae of capillaries and small venules [16]. Researchers postulated a perivascular exchange of ISF and CSF at the level of the capillary basal lamina consisting of a “sleeve-like macromolecular meshwork between the capillary endothelium and the surrounding end feet of astrocytes.” Neuropathologists refer to this structure as the glial limitans perivascularis.

### The “Glymphatic Pathway” is Formally Named

Using in vivo two-photon imaging in anesthetized mice, along with histology and radiotracer studies, Iliff et al. [2] provided further clarification about a “brain-wide pathway for fluid transport,” which they termed the “glymphatic pathway.” Intrathecal CSF tracer studies in vivo show rapid movement along arteries and arterioles, but the spaces around the veins and venules are not filled [2]. The perivascular space around the arterioles progressively narrows and becomes contiguous with the basal lamina (Fig. 2). This extracellular matrix is a porous gel composed of type IV collagen, fibronectin, laminin, and proteoglycans.

How does CSF entering brain parenchyma from the perivascular spaces, and how does the basal lamina cross over the astrocytic end-feet that line the perivascular space and the basal lamina? Illiff et al. [2] keyed in on the glial lining of cerebral vasculature by astrocytic end-feet, which is an important component of the neurovascular unit. They proposed that subarachnoid CSF enters brain interstitium by transglial movement involving the astrocytic end-feet that line the microvasculature, formally known as the glial limitans perivascularis. Narrow clefts between glial end-feet serve as a molecular sieve, as solutes pass through these endplates in a size- and molecular-structure-dependent manner. The aquaporin 4 channel (AQP4) is highly expressed on the astroglial end-feet. AQP4 plays an important role in CSF/ISF exchange, as AQP4-null mice have a 70% reduction in clearance of interstitial solutes. The current model is that CSF, ISF, and soluble molecules are then cleared along the perivascular spaces of draining veins, but this remains an area of active investigation.

In addition to the circulation of CSF and ISF, the glymphatic system plays a major role in removing waste products. For example, the glymphatic pathway clears out fluorescent-tagged and radiolabeled β-amyloid [2]. This finding has clinical significance, as accumulation of interstitial β-amyloid increases amyloid plaque burden and risk of Alzheimer disease. Perivascular accumulation of amyloid causes amyloid angiopathy, a leading cause of lobar cerebral hemorrhage in the elderly. We suggest these additional review articles for readers interested in more in-depth discussion of the basic science [19–23].

### Confirmation of the Glymphatic System in Humans

Because intrathecal gadolinium (Gad) does not cross the intact blood–brain barrier, it is a useful CSF tracer and can be noninvasively monitored by using serial magnetic resonance imaging (MRI) [24]. There is limited safety information for intrathecal Gad in humans, and it is not US Food and Drug Administration approved for this use. It appears well tolerated at small doses, but neurotoxicity has been observed at higher doses [25]. Ringstad et al. [26] used gadobutrol as a CSF tracer after special permission was granted by the National Medicine Agency of Norway. Under fluoroscopic lumbar puncture, intrathecal injection of 0.5 ml of 1.0 mmol/ml gadobutrol (Gadovist; Bayer, Leverkusen, Germany) was performed in near-normal control subjects with suspected spontaneous intracranial hypotension to look for spinal CSF leakages. Serial brain T1-weighted MRI studies were completed at multiple time points during the initial 24-h period, then at 48 h and 4 weeks.

To our knowledge, this study is the first confirmation of the glymphatic pathway in humans. The temporal and spatial pattern for CSF tracer distribution is remarkably consistent with the observations of Dandy. Entry of gadobutrol was most prominent in tissue adjacent to major cerebral arteries. Cerebellar uptake is also quite robust. CSF tracer was observed throughout the cerebral mantle (Fig. 1, middle row), peaking around 24–48 h (Fig. 1, bottom row) and then completely clearing after 4 weeks. Confirmation of complete clearance of intrathecal gadobutrol is an important finding regarding patient safety. The routes for clearance of the intrathecal gadobutrol CSF tracer from the brain and cranium were not determined in this study. Earlier work by this group documented intrathecal gadobutrol drainage in humans into cervical lymph nodes [27]. Additional studies are needed to confirm that these methods and observations are reliable and reproducible.

### Revival of Research into Weed’s Accessory and Lymphatic Pathways of CSF Drainage

**Fig. 3:**
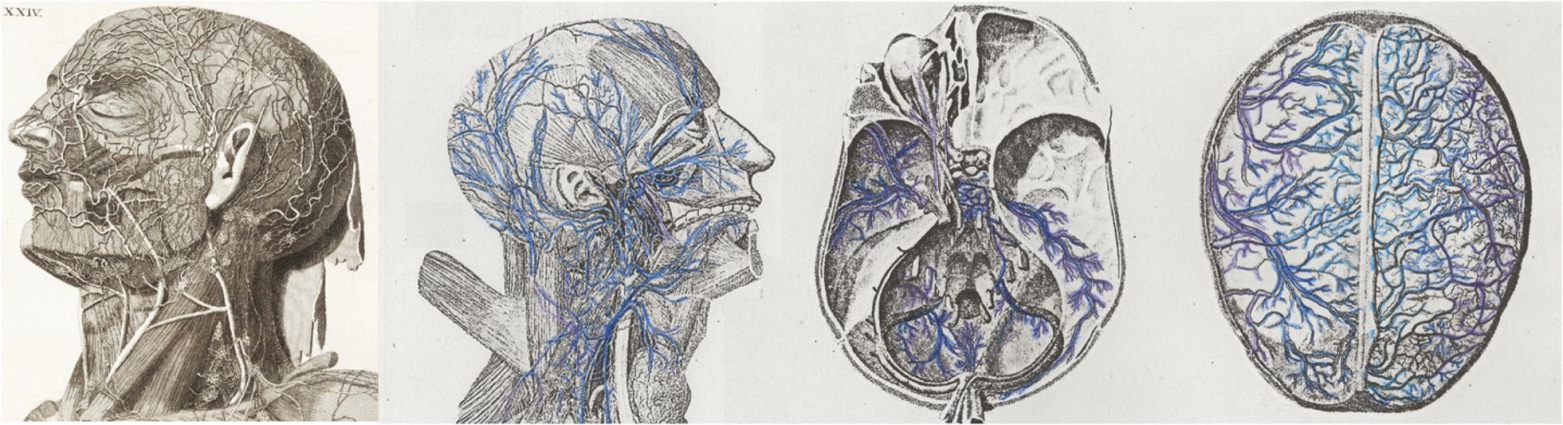
After cerebrospinal fluid (CSF) tracers penetrate deep into brain tissue along the glymphatic pathway, much of the tracer is cleared by cranial and cervical lymphatic vessels. In 1787 [28], using a right angle tubular needle and mercury, Mascagni published beautiful anatomical studies of these vessels (historical engravings with vessels colorized in blue and purple)

Cranial and spinal lymphatics play an important and unappreciated role in drainage of CSF and waste products from the central nervous system (CNS). Mascagni [28] used a mercury-injection method and a right angle tubular needle to study the lymphatic system in humans and published his work in 1787 (Fig. 3). Koh et al. [29] summarized later publications, dating from 1869 to 2005, describing the lymphatic system using a variety of CSF tracers and animal models with remarkably consistent findings, and the lymphatic system has been reported in nonhuman primates and humans [30].

Although Weed’s arachnoid villi have stolen the limelight for CSF absorption, the pendulum of recent evidence is ironically shifting toward Weed’s “accessory or lymphatic pathway of (cerebrospinal fluid) absorption.” In rabbits, 50% of ^131^I albumin microinjected into the caudate nucleus drains out along perivascular spaces into the olfactory lobe, crosses over the submucous space of the nasal epithelium, and is cleared by deep cervical lymphatics [31]. In sheep, 40% of CSF is cleared by the cervical lymphatics. Lymphatics are also found along spinal nerve roots.

In 2015, Louveau et al. [32] were researching how T cells traffic through the meninges. They discovered lymphatic vessels in mice around the eyes and olfactory bulbs that extend and align along the dural sinuses and then drain out into deep cervical lymph nodes. In formalin-fixed human dura, they found robust immunohistochemical staining with the lymphatic endothelial cell marker, Lyve 1. They concluded that the meningeal lymphatics “may represent the second step in the drainage of the ISF from the brain parenchyma…through the recently discovered glymphatic system.” Also in 2015, Aspelund et al. [33] reported similar findings while investigating cerebral lymphatic drainage in mice using a variety of lymphatic markers. Interestingly, structural features of dural lymphatic vessels differ by cranial site [34]. The basal lymphatic vessels have valves and adjacent capillaries and are very active in clearance of CSF solutes.

Recent studies conducted in primates and humans by using intrathecal gadobutrol and high-resolution T2-weighted fluid-attenuated inversion recovery and T1-weighted black-blood MRI sequences can resolve lymphatic drainage separate from venous drainage [27]. Immunohistochemical stains in human tissue have confirmed lymphatic vessels in the dura mater that travel alongside venous sinuses and branches of the middle meningeal artery.

### Incorporation of the Glymphatic System into CSF Circulation is a Paradigm Shift

With so much evidence confirming bulk flow of CSF, ISF, and macromolecules through a poroelastic CNS, it is time to reconceptualize brain fluid circulation. Before we focus on the clinical implications of the glymphatic system, we suggest a simple analogy using waterfalls to illustrate a fundamental difference between the classic model of CSF circulation and a revised model that incorporates the glymphatic system.

**Fig. 4:**
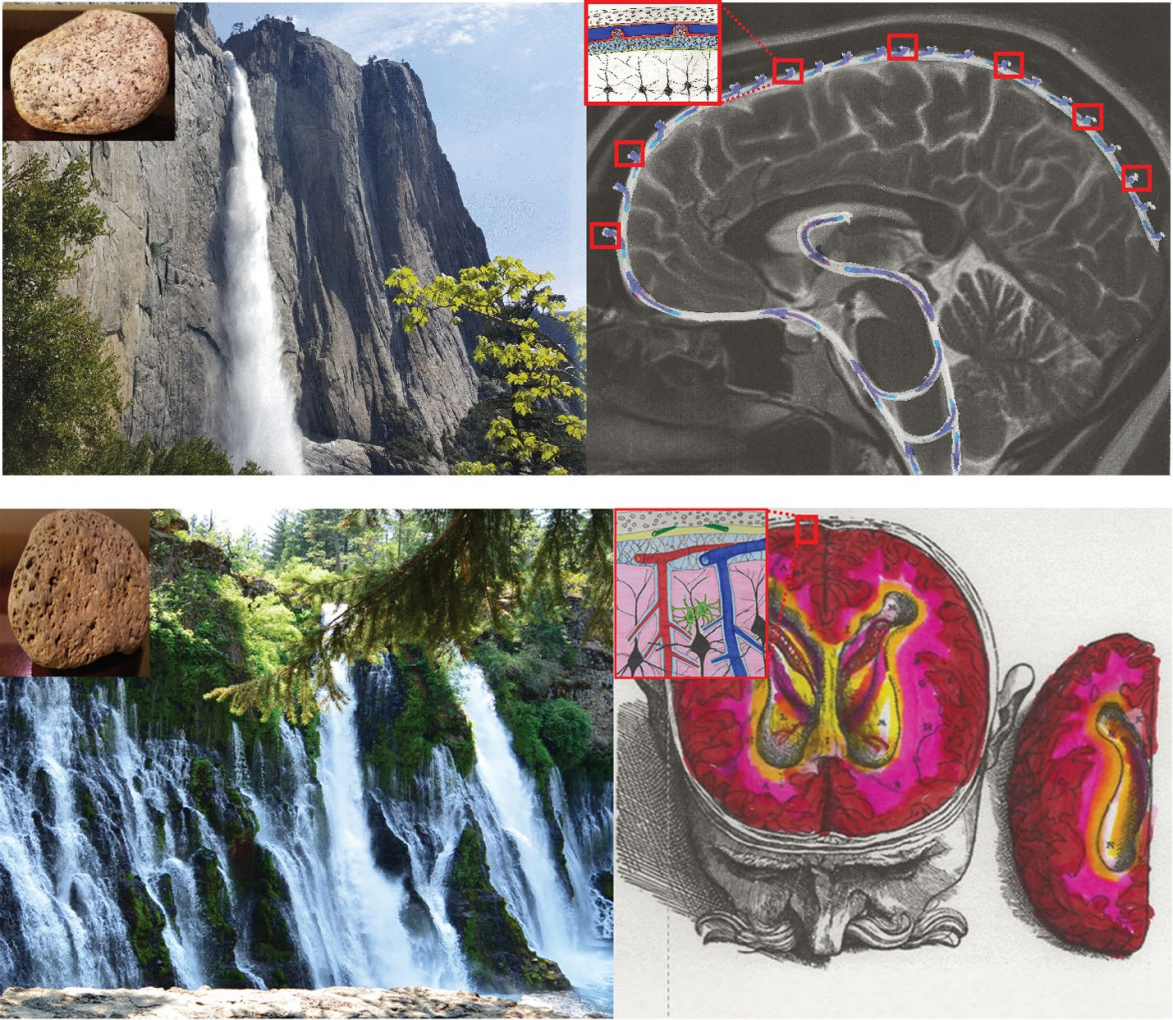
Analogy using waterfalls to compare the classic model of cerebrospinal fluid (CSF) circulation with a revised model that includes the glymphatic system. Yosemite Falls pours down the impervious, steep granite (inset) wall and flows into the Merced River in the valley (top row, left). The flow of water is analogous to the classic model of CSF, which explains how CSF travels through the ventricular system into the subarachnoid space and is absorbed into arachnoid villi (top row, right). The appearance of Burney Falls is strikingly different from that of Yosemite Falls (bottom row, left). Water pours over the lip of the falls, but there is a dual source of water coming directly out of the face of the falls. Burney Falls is fed by snowmelt that collects on the nearby volcanic peak of Mount Lassen and feeds Burney Creek and underground aquifers. Water travels overland but also underground through the porous and fractured basalt (inset). The flow of water both over and through the porous basalt substrate of Burney Falls is analogous to the glymphatic model of brain fluid, in which CSF moves through the ventricles and subarachnoid space but also by bulk flow through the perivascular spaces of the glymphatic system

Readers may recognize Yosemite Falls, which cascades over the steep granite walls for 2425 ft (739 m) into Yosemite Valley (Fig. 4, top row, left). Just 300 miles (482 km) north is Burney Falls, located just outside Lassen National Park (Fig. 4, bottom row, left), which awe-struck President Theodore Roosevelt (US president from 1901 to 1909) declared the “Eighth Wonder of the World.” The key to understanding how these two falls differ is to analyze their geology. Yosemite Falls pour over relatively impervious granite, whereas Burney Falls travels over fractured and porous basalt. Burney Falls is fed by dual sources. Snowmelt from nearby Mount Lassen (3187 m) renews the aquifer that feeds Burney Creek by an artesian spring and also supplies subterranean water that bursts through the porous basalt face of Burney Falls.

The flow of Yosemite Falls over impervious granite is analogous to the classic model of CSF circulation, in which CSF flows through ventricles and the subarachnoid space but does not incorporate bulk flow of CSF through the brain. The flow of water both over and through the porous basalt substrate of Burney Falls is analogous to the glymphatic model, in which CSF moves through the ventricles and subarachnoid space but also percolates deep into the brain via the perivascular spaces.

## Clinical Implications of Glymphedema Due to Inhibition of the Glymphatic Pathway

Our review of the clinical implications of the glymphatic system will place emphasis on diseases diagnosed and managed by neurocritical care and neurosurgical specialists.

### Hydrocephalus and Hygromas After Decompressive Craniectomy

Robust clinical and basic science research demonstrates that decompressive craniectomy reduces glymphatic drainage, which can lead to glymphedema. We previously defined glymphedema as abnormal craniospinal fluid collections due to impaired glymphatic drainage. These abnormal collections also signal that waste products are not being cleared either. Delayed hygromas and hydrocephalus are common after decompressive craniectomy, particularly when the indication for surgery is refractory elevated intracranial pressure (ICP) caused by severe traumatic brain injury (TBI).

We recently reviewed the extensive literature and our personal observations [36]. We proposed that these abnormal accumulations are due to the following: (1) replacement of the rigid cranial vault with a cranium modified with a large and highly compliant craniectomy site, (2) marked reduction in brain pulsatility and diminished ventricular squeezing due to dampened arterial pulsations, (3)impaired CSF circulation along the subarachnoid and perivascular spaces due to abnormal CSF hydrodynamics, (4) reduced clearance of ISF and solutes by the glymphatic system, and (5) redistribution of CSF from the subarachnoid space into the ventricles and the subdural and subgaleal compartments.

**Fig. 5:**
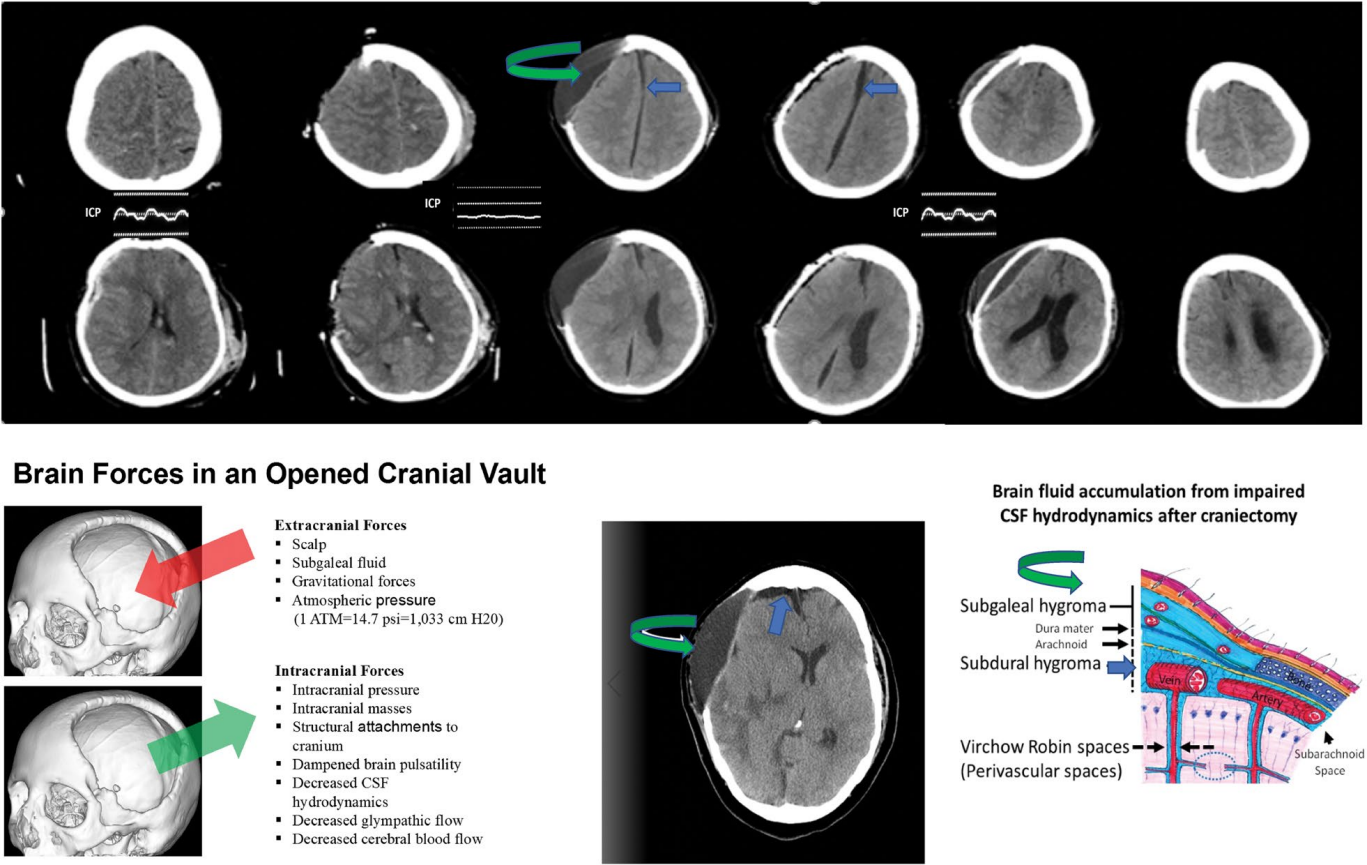
A temporal sequence with representative axial computed tomography scans of traumatic subdural hematoma, subarachnoid hemorrhage, and a cerebral contusion managed by craniectomy illustrating glymphedema manifesting as subgaleal and subdural hygromas (top panel). Following craniectomy, intracranial pressure (ICP) normalized with a flattened waveform, which is expected after craniectomy because of the large compliant cranial defect. A delayed large/tense subgaleal hematoma (green curved arrow) and a parafalcine hygroma (blue straight arrow) developed. Cranioplasty was performed, and the extra-axial collections resolved. A schematic of altered extracranial and intracranial forces following craniectomy (bottom left panel) and an anatomical schematic of glymphedema after craniectomy that develops because of decreased brain and arterial pulsatility, impaired cerebrospinal fluid (CSF) hydrodynamics, and reduced glymphatic clearance of fluid/waste solutes (bottom right panel) are shown. This caused hydrocephalus and subdural (green curved arrow) and subgaleal (blue straight arrow) hygromas

Clinical observations support this hypothesis. The ICP waveform is markedly dampened following craniectomy (Fig. 5) because of the large compliant cranial defect with diminished brain pulsatility [37]. Cerebral blood flow (CBF) is decreased, particularly ipsilateral to the craniectomy [38]. CSF flow in the aqueduct is stagnant because of diminished ventricular squeezing and brain pulsatility [39]. This reduces the normal forces that drive glymphatic circulation. Cranioplasty restores aqueductal CSF flow and also increases CBF [38, 39]. In many patients, cranioplasty without shunting can alleviate hydrocephalus and hygromas due to decompressive craniectomy [40].

Plog et al. [41] published confirmatory observations in mice that craniectomy profoundly inhibits the glymphatic pathway by decreasing arterial pulsatility and thereby reducing glymphatic CSF influx. The glymphatic dysfunction was accompanied by elevated astrocytic and microglial inflammatory responses. Cranioplasty restored glymphatic flow. They independently proposed the hypothesis that altered CSF hydrodynamics after craniectomy is due to “reduced glymphatic conductance of CSF into the brain parenchyma [that] leads to upstream accumulation, first within the subarachnoid space (subdural hygroma), and if severe and persistent enough, later within the ventricular system (communicating hydrocephalus).” Parentheticals emphasize the corresponding radiographic findings in patients.

### Subarachnoid Hemorrhage

**Fig. 6:**
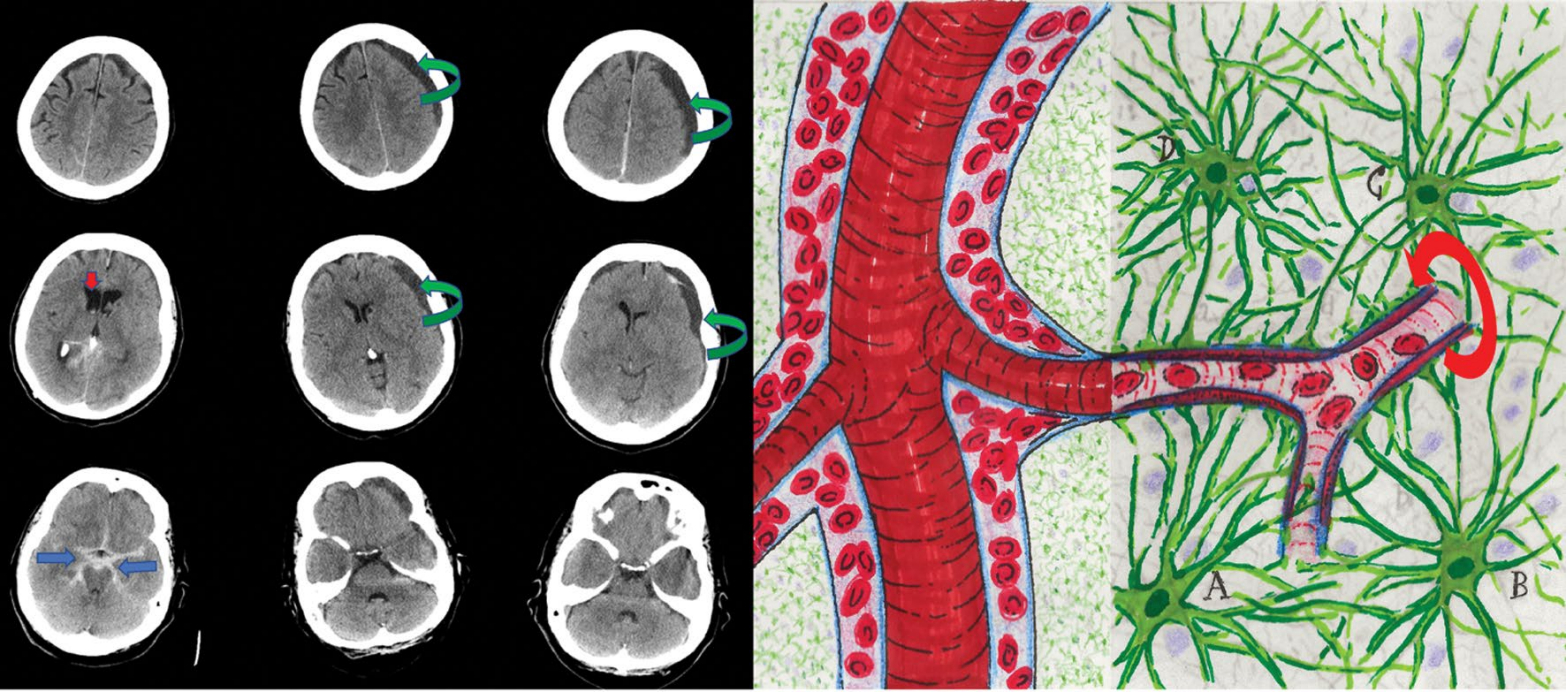
The initial pattern of aneurysmal subarachnoid hemorrhage (SAH) is along the basal cisterns (blue arrows) and the subarachnoid space adjacent to the circle of Willis and its major arterial branches, as seen on these representative axial computed tomography images (left column). Impaired cerebrospinal fluid (CSF) circulation causing ventricular dilation (red arrow) occurs frequently. Acute blood products radiographically clear the subarachnoid space within a few days, but persistent abnormalities in CSF circulation and absorption are common (middle and right columns). This patient had progressively enlarging hygromas with mass effect that responded to delayed burr hole drainage. Animal models of SAH demonstrate that the blood in the subarachnoid space rapidly penetrates into brain tissue along perivascular spaces (Virchow–Robin spaces) (right figure), activating a cascade of events, including glymphedema with impaired clearance of waste products (including tau proteins), inflammatory responses in glia and microglia, cytokine production, deposition of degraded blood products and fibrinogen between the basal lamina and glial end-feet, microvascular spasm (left panel, curved red arrow), and neuronal apoptosis in the hippocampus

Following nontraumatic subarachnoid hemorrhage (SAH), hydrocephalus often develops rapidly, and hygromas may form too (Fig. 6). Although a common clinical explanation is that the blood in the subarachnoid space causes impaired CSF drainage from blocked arachnoid granulations, we now know that SAH severely disrupts the glymphatic system.

Luo et al. [42] observed the perivascular space of pial arteries using in vivo two-photon imaging in both wild-type mice and AQP4-knockout mice after cisternal injection of blood labeled with Fluorescein Isothiocyanate (FITC)-d2000 in a SAH animal model. Fluorescently labeled blood entered the perivascular space of the pial and penetrating arteries in less than 1 min. Other blood products, such as fibrinogen, ferritin, and heme oxygenase 1, were also found in the perivascular space. Microvascular spasm was triggered by the blood in the perivascular space. Experimental SAH triggered a robust inflammatory response. By day 7, they observed activated microglia and reactive astrocytes and increases in inflammatory markers, such as Toll-like receptor 4, tumor necrosis factor-α, interleukin-1β, and monocyte chemoattractant protein-1 (MCP1). Intrathecal tissue plasminogen activator infused after SAH improved clearance of the fluorescent CSF tracers from the perivascular space and diminished inflammatory responses. AQP4-knockout mice showed reduced entry of intrathecal blood into brain parenchyma, but neurologic deficits and delayed inflammatory responses were similar. Brain tissue biopsy specimens from patients with SAH undergoing craniotomy and aneurysm clipping were analyzed, and perivascular red blood cells were found adjacent to small arteries and penetrating arterioles (Fig. 6).

Intrathecal Gad studies using serial MRI in rodent and primate SAH models [43, 44] also demonstrate impaired glymphatic circulation. Following SAH, parenchymal penetration and clearance of the Gad CSF tracer is severely reduced. After experimental SAH and euthanasia, microscopic examination of the primate brain tissue confirmed fibrinogen staining between the outer wall of the perivascular space and the astrocytic feet (glial limitans perivascularis).

Pu et al. [45] confirmed impaired glymphatic circulation and meningeal lymphatic drainage in a mouse SAH model using a variety of methods. They provided an elegant summary of the downstream effects of SAH on the glymphatic system. Key findings were (1) reduced CSF tracer movement into brain parenchyma along perivascular spaces, (2) reduced CSF tracer in meningeal and cervical lymphatics, (3) microvascular spasm, (4) glial cell activation, (5) accumulations of tau proteins, (6) neuronal apoptosis in the hippocampus, and (7) glymphedema confirmed by measuring an increase in brain water content 1 day after SAH.

These consistent and reproducible observations of glymphatic dysfunction caused by SAH close major gaps in our understanding of SAH pathophysiology. For example, it is known that regional hypoperfusion after SAH does not always correlate with cerebral vasospasm in medium and large arteries supplying these oligemic brain regions on the basis of neurovascular imaging, such as cerebral angiography, computed tomography angiography, or transcranial Doppler. Remarkably, approximately one fourth of patients with regional cerebral hypoperfusion measured by Positron-emission tomography CBF in fact had no signs of vasospasm on catheter angiography [46]. The microvascular spasm observed in response to blood and inflammation in the perivascular spaces explains how regional hypoperfusion can occur despite the absence of macroscopic vasospasm readily detected by standard neurovascular imaging methods.

Incorporating glymphatic dysfunction into the pathophysiology of SAH points to new therapeutic approaches. Although hemodynamic augmentation is standard treatment for patients with SAH with symptomatic CNS vasospasm, pressors also boost the arterial pulsatility, which is a key force driving glymphatic flow. For example, systemic dobutamine improved glymphatic function in mice [47]. Whether hemodynamic augmentation improves glymphatic dysfunction in SAH animal models and patients with SAH requires further study. Intrathecal tissue plasminogen activator reduces blood and blood products in the perivascular space and improves glymphatic function in SAH animal models [26, 42]. In a meta-analysis and review of 21 published studies encompassing 1373 patients and 9 randomized controlled trials [48], intracisternal fibrinolysis decreased the odds for poor neurologic outcomes and delayed ischemic neurologic deficits, chronic hydrocephalus, and mortality.

We predict that a multifaceted “cocktail” approach may be needed to accelerate clearance of blood products, reduce the inflammatory response, and temporally augment the glymphatic circulation. The glymphatic system fluctuates on a circadian rhythm and is most active during deep non-rapid-eye-movement sleep [49, 50]. Clinicians should consider benefits/risks of frequent neuro-checks, which interrupt sleep and decrease frequency for patients safely past the initial acute phase of care. When selecting sedatives for patients with SAH, dexmedetomidine, a selective α-2-adrenergic agonist, has theoretical benefits on the glymphatic system. It improved the glymphatic clearance of intrathecal oxycodone and naloxone [51]. The proposed mechanism is inhibition of the locus-coeruleus-mediated norepinephrine release through the ascending cortical projections causing a non-rapid-eye-movement sleep-like state with slow-wave electroencephalographic oscillations.

### Normal-Pressure Hydrocephalus

Normal-pressure hydrocephalus is a subtype of hydrocephalus that shows symptomatic ventriculomegaly without elevated ICP readings [11–13]. Variable degrees of clinical improvement are observed after placement of programmable ventricular shunts. Although the causes of NPH are not fully understood, recent work using magnetic resonance elastography measured abnormal viscoelastic properties in patients with NPH compared with age-matched controls [52]. The authors used the rheological spring-pot viscoelastic model, which has two key parameters. The shear modulus parameter (*m*) is a measure of global brain stiffness and was decreased in patients with NPH. The second parameter (*a*) is low in simple gels and increased in biologic tissue with three-dimensional structures, such as nervous tissue. Patients with NPH had lower *a* values compared with controls, which is consistent with the greater ventricular compliance and the lower-pressure shunt settings needed for patients with NPH. After shunting, repeat magnetic resonance elastography showed near normalization of *a* but no effect on global brain stiffness, *m*.

Delayed glymphatic clearance has been directly measured in patients with NPH. Following lumbar intrathecal gadobutrol injection as a CSF tracer, Ringstad et al. [53] completed serial MRI over 24 h in patients diagnosed with NPH (*n* = 15). ICP was also monitored. All patients were kept in a supine position during the study period because glymphatic clearance is position dependent. They observed (1) delayed glymphatic clearance of intrathecal Gad, (2) retrograde filling of the ventricular system, and (3) transependymal enhancement. Entorhinal cortex atrophy and medial temporal lobe atrophy were increased in patients with NPH compared with controls.

On the basis of animal studies showing the role of the glymphatic system in clearing macromolecules, such as soluble amyloid, from the brain [2], the reduced glymphatic clearance may contribute to a buildup of neurotoxic molecules, such as amyloid and tau proteins, in patients with NPH. These findings may account for the delayed cognitive and functional decline commonly seen in shunted patients with NPH after a honeymoon period of clinical improvement. Because AQP4 expression at astrocytic end-feet lining brain blood vessels is fundamental for normal glymphatic clearance [54], Hasan-Olive et al. [55] conducted studies of AQP4 in brain biopsy specimens from reference (*n* = 12) patients and patients with NPH (*n* = 30). Electron microscopy using immunogold cytochemistry found that the perivascular density of AQP4 within astrocytic end-feet was reduced in NPH patients vs. controls.

### Ischemic Stroke Edema

Acute edema that develops after cerebral ischemia has traditionally be attributed to cytotoxic edema related to cellular energy depletion, failure of membrane integrity, and cellular swelling. Evidence is building that impaired glymphatic clearance is also a contributor. Acute carotid ligation [47] slows the ipsilateral glymphatic pathway measured by cortical influx of a fluorescent CSF tracer by 28% by reducing the driving force from arterial pulsations. Gaberel et al. [26] studied the glymphatic system in mice after embolic ischemic stroke using intrathecal Gad and MRI and also histofluorescence after intracisternal sodium fluorescein. They found decreased glymphatic clearance at 4 h and restoration at 24 h. They attribute the transient nature of the glymphedema to the spontaneous arterial recanalization that is common with this animal stroke model.

In a middle cerebral artery occlusion model in mice, Mestre et al. [56] determined that the earliest edema after ischemic stroke is related to CSF influx from the subarachnoid space into perivascular spaces. The sequence of events that triggers this influx is not immediately intuitive. First, the cerebral ischemia causes spreading cortical depolarizations causing arterial vasoconstriction. The vasoconstriction increases the capacity of the perivascular space to accommodate influx of CSF from the adjoining subarachnoid space. Histologic examination in mice and human brain tissue after cerebral ischemia showed increased fluid (glymphedema) in brain tissue closest to the subarachnoid space and ventricles.

Common clinical practices to manage cerebral edema after hemispheric stroke include hyperosmolar treatments and decompressive craniectomy [57]. We are not aware of clinical or animal studies investigating the effects of hyperosmolar treatments on the glymphatic pathway in controls or following ischemic stroke. Although prophylactic decompressive craniectomy lowers stroke mortality, we now know that it carries the cost of impaired glymphatic clearance in addition to other postsurgical complications [58].

### Traumatic Brain Injury

By using a hit-and-run model for TBI in mice (closed skull cortical impact), impaired CSF fluorescent tracer glymphatic clearance was observed ipsilateral to the closed head injury but also to a lesser degree in the contralateral side [59]. This finding was associated with a loss of AQP4 polarization at astrocytic end-feet. Brain injections of radiolabeled solutes, such as mannitol and inulin, showed impaired clearance. Given concerns about chronic tau encephalopathy after head trauma, P-tau immunoreactivity was measured in this TBI model. In wild-type mice, mild elevations were seen, but AQP4-knockout mice had pronounced P-tau cortical immunoreactivity accompanied by neuroinflammation and axonal degeneration.

The researchers proposed that TBI leads to chronic loss of AQP4 polarization at astrocytic end-feet, which impairs glymphatic clearance of interstitial tau. This promotes tau aggregation and associated neuroinflammation and neurodegeneration, which are hallmarks of chronic tau encephalopathy. TBI also reduces meningeal and deep cervical lymphatic drainage in animal models [60]. Clinical studies are needed to confirm these observations.

### Hepatic Encephalopathy

Cerebral edema is an ominous complication of liver failure. By using a rat model of liver disease using bile duct ligation [61], MRI was obtained following intrathecal Gad as a CSF tracer in a small animal study (*n* = 8). Microinjections of Gad into brain were also studied. Glymphatic clearance was decreased in certain brain regions, such as the olfactory bulb, hippocampus, and prefrontal cortex. AQP4 immunofluorescence was lower in the olfactory bulb and prefrontal cortex after bile duct ligation compared to sham-operated animals, which could provide a mechanism for the regional reductions in glymphatic clearance. More experiments are needed to replicate these results and confirm them in clinical studies.

## Additional Candidate Diseases and Future Directions

We predict that the glymphatic system is disrupted in many other diseases commonly managed by neurocritical care and neurosurgery. For example, extrinsic and intrinsic brain tumors cause local mass effects on the subarachnoid and perivascular spaces that would be expected to cause regional impairment in glymphatic circulation. Local surgical disruption of dural lymphatic drainage may occur as well, particularly with extra-axial tumors, such as meningiomas. Surgery is also expected to cause a transient disruption in the glymphatic pathway by extrapolation from studies of decompressive craniectomy and cranioplasty. Cranial radiation damages blood vessels, often in a delayed fashion, so it is likely that lymphatic vessels are also injured. Impaired glymphatic clearance and glymphedema may contribute to delayed pseudomeningocele formation and CSF leaks after skull base surgeries. This may explain why primary dural closure and complete calvarial reconstructions lower risks of these surgical complications, as these extra steps will restore cranial rigidity and brain pulsations that are vital to glymphatic function [62].

**Fig. 7:**
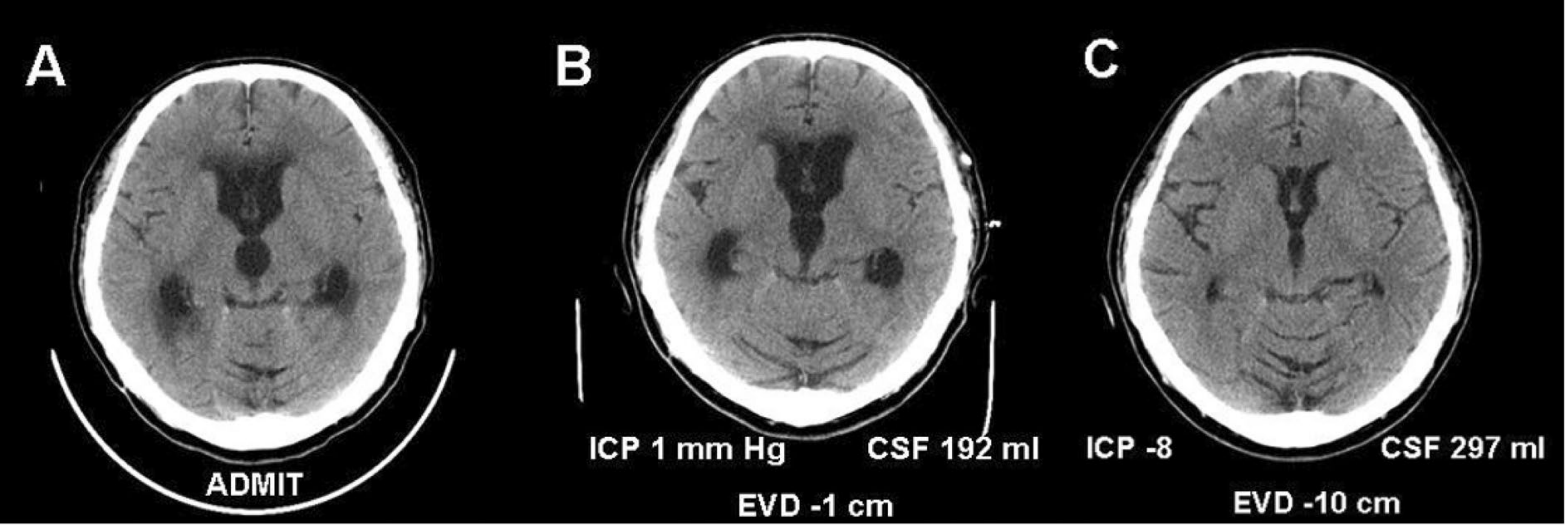
Low-pressure hydrocephalus is a rare variant of communicating hydrocephalus in which traditional shunting procedures are initially not successful. Subarachnoid hemorrhage (SAH) is a common cause, so this condition may represent an important link between studies investigating glymphatic dysfunction in patients with SAH and normal-pressure hydrocephalus. We had previously described this as a “boggy brain state” [63] but now suspect that this represents glymphedema. **a** Communicating hydrocephalus in a patient with disseminated coccidioidomycosis. **b** After ventricular drain insertion, there was marginal improvement in ventriculomegaly, with drain at 1 cm below the external auditory meatus (EAC) and daily cerebrospinal fluid (CSF) output of 192 ml. **c** With ventricular drainage 10 cm below the EAC, subatmospheric pressure drainage was achieved (− 8-mm Hg average daily intracranial pressure [ICP]), and greater daily CSF output was drained (297 ml) effectively, causing “brain diuresis.” Negative-pressure (also termed “subzero”) drainage for a prolonged period of time partially restores the biophysical properties of the brain so that eventually a ventricular shunt can be internalized (ventriculoperitoneal or ventriculopleural shunt). It is likely that the excess fluid that is mobilized is a combination of CSF and interstitial fluid. Research is needed to investigate whether the negative-pressure drainage reverses flow in the perivascular spaces and restores glymphatic function *EVD* external ventricular drain

Because SAH causes a chemical meningitis that adversely affects the glymphatic system, it is likely that meningeal inflammation due to infections and malignancies will also adversely affect it. Our group has investigated low-pressure hydrocephalus (LPH) triggered by SAH and chronic CNS infections, such as coccidioidomycosis [63]. Our findings and others indicate that LPH is caused by biophysical changes in the brain from a spectrum of initial insults. We speculate that LPH may represent a more extreme impairment of the glymphatic system than observed in patients with NPH or seen with SAH animal models. It will be intriguing to study whether the beneficial effects of negative-pressure CSF drainage (also called subatmospheric or subzero drainage) are an important clue to restore glymphatic function. We previously observed that this approach will mobilize CSF/ISF from the “boggy brain” parenchyma and perivascular spaces (Fig. 7), basically “brain diuresis.”

The glymphatic system may provide insights into other poorly understood diseases characterized by cerebral edema and altered CSF circulation. Examples include posterior reversible encephalopathy syndrome, high-altitude cerebral edema [64], reversible cerebral vasoconstriction syndrome, and idiopathic intracranial hypertension [65, 66].

With rising longevity and declining birth rates in many countries, older patients are now heavily represented in neurocritical care and neurosurgery practices. Therefore, we would like to make readers aware that the glymphatic system declines with aging [67, 68], dementia [69], and chronic diseases, such as diabetes [67].

### Current and Emerging Methods to Measure Glymphatic Function

Finding methods to safely and reliably measure glymphatic function in patients is a key challenge to translational research of the glymphatic system. Two-photon microscopy provides high spatial and temporal resolution but is not practical in patients because of the need for continuous monitoring through a small craniotomy and the limited region of the brain that can be studied. Postmortem analysis of the perivascular distribution of intrathecal injection of CSF tracers using light and electron microscopy has provided convincing evidence for the glymphatic system in human subjects, but the clinical utility is limited to surgical specimens and autopsy material.

To date, the most successful method to measure the glymphatic system in patients is with MRI [28, 53, 70–72]. We previously reviewed the use of serial MRI after intrathecal Gad (also called dynamic contrast-enhanced MRI) to study the glymphatic system in near-normal control subjects and patients with NPH [28, 53]. Limitations are that intrathecal use of Gad is not US Food and Drug Administration approved, serial MRI acquisition and image processing are labor intensive, dose-related neurotoxicity can occur [27], and CSF tracer administration is invasive. This approach remains fruitful, and impaired glymphatic and meningeal lymphatic drainage associated with aging in humans was recently reported by using this method [73].

Redistribution of intravenous Gad into CSF and the perivascular space can be detected in humans with MRI, but clinical utility is uncertain [74]. Preliminary reports suggest that diffusion tensor MRI may also be a useful noninvasive way to study perivascular flow [75, 76]. Positron-emission tomography–computed tomography by using intrathecal CSF tracers (2-deoxy-2-[^18^F]fluoroglucose) could be an alternative to intrathecal Gad/MRI and has been used to monitor glymphatic flow in a small rodent study [77].

## Conclusions

Research into the glymphatic system has reached an inflection point with steep trajectory, but key elements were identified more than a century ago. This literature provides robust support for the glymphatic system, which promotes bulk flow of CSF, ISF, and solutes throughout the brain parenchyma. Evidence is building that failure of the glymphatic system causes glymphedema, which has important clinical relevance for neurocritical care and neurosurgery specialists. We encourage readers to visit other clinical reviews that explore how impaired clearance of cerebral waste products may play a pivotal role in brain aging and neurodegenerative diseases [58–61].

Now is the time to embrace a paradigm shift from the traditional model of CSF circulation to a revised model that incorporates the glymphatic pathway and lymphatic clearance. Once the field settles on safe and approved CSF tracers readily tracked by MRI, this will remove a major bottleneck for clinical research. We are optimistic that recent and novel breakthroughs will inspire new therapeutic approaches to recognize, reverse, and restore glymphatic dysfunction and to leverage this pathway to deliver brain-wide therapeutics.

## Abbreviations

AQP4: Aquaporin 4 channel
CBF: Cerebral blood flow
EAC: External auditory meatus
ECS: Extracellular space
Gad: Gadolinium
HRP: Horseradish peroxidase
ISF: Interstitial fluid
IT: Intrathecal
LPH: Low pressure hydrocephalus
NPH: Normal pressure hydrocephalus
REM: Rapid eye movement
SAH: Subarachnoid hemorrhage
TBI: Traumatic brain injury
